# Measurement Instruments for Integration within Children and Young People Healthcare Systems and Networks: A Rapid Review of the International Literature

**DOI:** 10.5334/ijic.7028

**Published:** 2023-05-23

**Authors:** Zainab Dedat, Steven Hope, Dougal Hargreaves, Oliver Lloyd-Houldey, Dasha Nicholls, Steph Scott, Evgenia Stepanova, Carolyn Summerbell, Russell M. Viner, Frances Hillier-Brown

**Affiliations:** 1 Population, Policy & Practice Research & Teaching Department, UCL Great Ormond St. Institute of Child Health, London, UK; 2 Mohn Centre for Children’s Health & Wellbeing, School of Public Health, Imperial College London, UK; 3 Division of Psychiatry, Department of Brain Sciences, Imperial College London, UK; 4 Fuse –Centre for Translational Research in Public Health, UK; 5 Population Health Sciences Institute, Newcastle University, UK; 6 Department of Sport and Exercise Sciences, Durham University, UK

**Keywords:** integrated care, health care, measurement, children and young people, rapid review

## Abstract

**Introduction:**

Robust measures of integration are essential for assessment of the development, design and implementation of integration within healthcare systems. This review aimed to identify measurement instruments for integration within children and young people’s (CYP) healthcare systems (PROSPERO registration number CRD42021235383).

**Methods:**

We searched electronic databases (PubMED and Ovid Embase) using three main concepts: ‘(integrated care) AND (child population) AND (measurement)’, along with additional searches.

**Results:**

Fifteen studies describing 16 measurement instruments were eligible for inclusion. The majority of studies were conducted in the USA. There was a diversity of health conditions included in the studies. The most frequent type of assessment used was a questionnaire (11 identified), but interviews, patient data and healthcare records, and focus groups were also used. Integration outcomes assessed were quality of care coordination, quality of collaboration, continuity of care, completeness of care, structure of care, quality of communication, and local implementation of integrated care.

**Conclusion:**

A variety of instruments for the measurement of integration within CYP healthcare systems were identified. Further work on the standardisation of integrated care measures would be valuable; however, it is important that instruments and measures meet the needs of specific settings, populations and conditions being studied.

## Introduction

Integration has become increasingly important in healthcare worldwide, enabling greater efficiencies, improved patient experiences and outcomes, reduced fragmentation of services and better coordination and continuity of care [1,2]. Integrated care is viewed as mechanism for addressing long-term and complex medical conditions, as demand on healthcare systems increase [3]. This is illustrated in England where a focus on integrated care has been on the National Health Service (NHS) agenda since the 1980s [1], although it was recognised as early as 1959 [4]. More recently, the 2019 NHS Long-Term Plan proposed integrated care systems (ICSs) as central to improving healthcare [2]. In April 2021, a total of 42 ICSs footprints were rolled out across England, bringing together in local areas all parts of the NHS and partners in social care and education, with an aim to “redesign care and improve population health, creating shared leadership and action” [5]. The burden of non-communicable disease among children and young people (CYP; aged 0–17 years) is a global challenge [6,7]. Alongside the implementation of ICSs, the NHS Long-Term Plan has a key focus on CYP, and the importance of developing CYP healthcare and network systems based on the principles of prevention and early intervention [2].

While integrated care is a commonly used concept in healthcare, the term lacks precision, with over 175 definitions [8]. According to a widely-accepted definition by Shaw, Rosen and Rumbold [2], “integrated care is an organising principle for care delivery with the aim of achieving improved patient care through better coordination of services provided” (p.7). Previous literature exploring adult healthcare systems has highlighted key elements of integrated care, including engaging and empowering people and communities, population-oriented strategies, care-coordination, patient focus, governance structure and organisational culture and leadership [9,10,11]. While many of these elements are important in integrating health services for CYP, this age group presents some distinct challenges and opportunities [7,12]. These include the challenge of changing physical and cognitive developmental needs within a relatively short time period and subsequent variation in caregiver involvement, along with increasing complex medical needs. Opportunities include medical and technical advancements, and improved data sharing systems. Integrated care aligns with the principles of the United Nations Convention on the Rights of the Child that state that CYP have the right to good quality health care, along with the right to information about their health and a say in how they get this.

Measuring integration is essential to effectively monitor the development, design and implementation of integrated healthcare systems and networks [13], such as those proposed in England’s NHS Long Term plan. Alongside this, it is also important to understand which integration outcomes are most useful to assess and there is a need for clearer understanding of components of integrated care for CYP, that are often complex and overlapping [7]. A separate review of components of integrated care for CYP conducted in parallel with this one has identified 23 distinct components that are influential for healthcare integration (Stepanova et al., unpublished). Each component is linked to three higher level domains of integration, including collaboration, coordination and co-location and is grouped in accordance with its area of focus (service users, staff or system). The ability to quantify different integration components and domains may help to understand where integration is being achieved, and where barriers to full integration can be addressed. Measurement of integration might occur at different levels and from perspectives of different stakeholders (e.g. CYP, care givers, and healthcare and other professionals).

Within adult healthcare systems and networks, a number of instruments have been developed and used to measure levels and components of integration [11,13,14,15]. However, fewer instruments have been published for CYP measurement. An earlier systematic review found a small number of standardised, validated instruments for the evaluation of integration, with no mention of measurement within CYP healthcare systems and networks [8]. A second review identified 23 instruments developed to measure the level of integrated care delivery in healthcare systems, however only one of these measurement instruments focused on families and young children [14]. A third systematic review identified 96 measurement instruments for integration, of which CYP were the target population for 21 instruments; however, these instruments only evaluated changes in coordination needs, rather than coordination itself [10].

There is a need to update the evidence-base on instruments for the measurement of integration within CYP healthcare systems. To our knowledge, no previous review has focused on identifying instruments for the measurement of integration within healthcare systems solely for CYP; therefore, the purpose of this rapid review is to identify and assess instruments designed to measure integration within CYP healthcare systems and networks.

## Methods

We conducted a rapid review following the guidance from Cochrane Rapid Reviews Methods Group [16] and reported our findings based on the Preferred Reporting Items for Systematic Reviews and Meta-Analyses Statement [17]. The review was registered with the PROSPERO registry of systematic reviews (registration number CRD42021235383). Rapid reviews are time-efficient, effective, streamlined, pragmatic methods to synthesis previous findings and evidence [18]. In this case, a rapid review was conducted as there was a narrow research question and a short-time frame in which to complete the review. The review also followed guidance set by the COnsensus-based Standards for the selection of health Measurement Instruments (COSMIN) on reviewing and reporting of health measurement instruments [19].

### Eligibility criteria

We used the PICO criteria in establishing eligibility criteria:

- Population (P) – Children and young people (less than 18 years old)
- Intervention (I) – Any integrated healthcare system for CYP, including collaborative care models; shared-care and multidisciplinary care; information sharing care models
- Comparator (C) – No comparator required (NA)
- Outcome (O) – Level of integration within CYP healthcare systems (as defined within the included studies)

We considered any study which described the development or evaluation (e.g., reliability, validity) of instruments designed to measure integration within a CYP healthcare system. We limited the search to studies in English.

### Literature search

We searched electronic databases (PubMED and Ovid Embase) from their inception date to March 2022. We used three main concepts as the basis of our search: ‘(integrated care) AND (child population) AND (measurement)’, and were guided by a search strategy used in a previous systematic review that we had identified [15]. Additional material was searched through the grey literature database Open Grey, alongside an online search using the search engine ‘Google’ (first 15 pages of results or until saturation was reached). Full details of the academic literature and grey literature search strategies can be found in Data file 1. Reference lists of all included studies and related systematic reviews were checked for additional studies.

### Study selection, data extraction and synthesis

Data were exported to the reference management software EndNote (Alphasoft Ltd, Luton, UK) which was used for all screening processes, including deduplication, title and abstract screening and full-text screening. All search results were screened by one reviewer and checked by a senior reviewer. Studies meeting the eligibility criteria were then fully assessed against the inclusion/exclusion criteria and accepted or rejected, as appropriate. Concordance was checked and any discrepancies were discussed and resolved between both reviewers.

We developed a standardised data extraction form (e.g., lead author, year published, country, study design, system type/characteristics, population characteristics, description of the measurement instrument, administration method, type of integration, and evaluation results (where reported)). The data extraction form was initially piloted by two reviewers using the full text of five of the included articles. Modifications were made where appropriate to improve the clarity of the form. All remaining data extractions were completed by one reviewer and checked by a senior reviewer. Due to the heterogeneity of included studies, we reported data using an initial descriptive synthesis, followed by a narrative synthesis.

The quality of studies evaluating the measurement properties of an instrument was assessed using the COSMIN Risk of Bias tool [20,21,22]. Where studies evaluated more than one measurement property of an instrument, separate quality appraisals were conducted based on methods related to each evaluation. For example, the same study might be rated as ‘very good’ for the evaluation of construct validity, but ‘inadequate’ for the evaluation of content validity.

## Results

### Included studies

**Figure 1:**
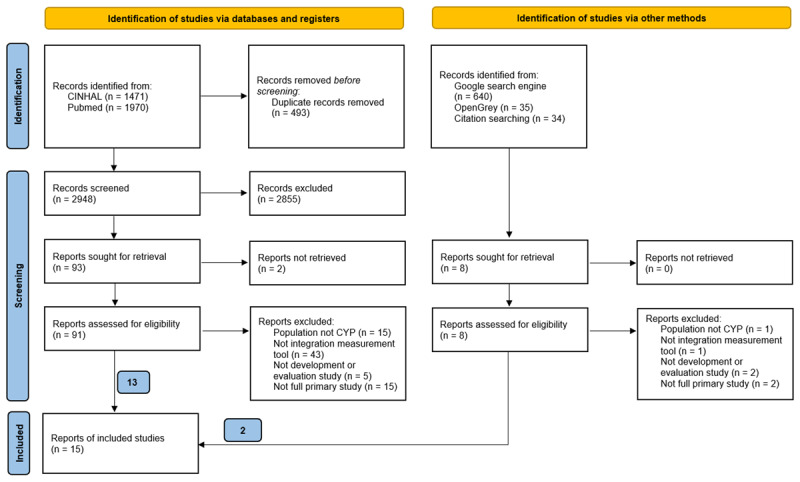
PRISMA diagram of the study selection process for instruments measuring integration within children and young people healthcare systems.

Following screening of 3657 articles and reports, 15 studies of 16 measurement instruments, were eligible for inclusion. The study selection process can be seen in Figure 1. Of the 15 studies, eight studies were conducted in the USA [23,24,25,26,27,28,29,30], three in the Netherlands [31,32,33], two in Canada [34,35], and one each in Brazil [36] and Japan [37]. All the studies were published post-2000. Eight studies measured integration within systems designed for children with medical complexity [23,24,25,26,28,33,35,37], two for mental health [29,34], and one each for ADHD [27], cerebral palsy [31], overweight and obesity [32], chronic conditions [30] and non-specific health condition [36]. Five studies described the development of a measurement instrument [25,29,31,35,36], and 14 studies included a type of evaluation of a measurement instrument: feasibility (n = 4) [23,26,32,34], reliability (n = 6) [27,29,30,34,35,37], and content (n = 3) [29,32,33], construct (n = 6) [24,27,28,29,30,37] and criterion (n = 1) [29] validity. A summary of included studies is presented in Data file 2 and the list of excluded studies presented in Data file 3.

### Measurement Instruments and integration outcomes

Table 1 shows the range of instruments used to measure integration and the different outcomes measured. The most frequent type of assessment used was a questionnaire, most commonly designed to be completed by healthcare and other professionals involved in the integrated healthcare system (n = 7) [27,32,34,35,36,37], or caregivers (n = 4) [25,26,29,30], with one questionnaire completed by CYP themselves [29]. Two instruments used service visit data (that could be identified easily by an administrator) as an indicator of quality of care coordination [23,24]. Two instruments used multiple data sources: a patient quality questionnaire, followed by in-depth interviews with a subset of patients and focus group meetings with involved professionals [31]; and patient case file evaluation, interviews with patients and caregivers, and meetings with associated professionals [33].

**Table 1:** Descriptions of instruments (n = 16) measuring integration within children and young people healthcare systems by outcomes measured.


MEASUREMENT INSTRUMENTS	TYPE OF MEASUREMENT INSTRUMENT	DESCRIPTION OF MEASUREMENT INSTRUMENTS

***Outcome: Quality of care coordination***		

Primary Care Assessment Tool (PCATool Brazil) [36]*† (Also measures Completeness of care and Structure of care)	Questionnaire (completed by HCPs)	55-item Likert-scale instrument Assesses the structure and process of the system by measuring longitudinally, accessibility, coordination, system information, integrality of care and other factors

Bice-Boxerman Continuity of Care index (indicator of quality of CC) [24]	Simple administration method (from visit records)	Measures the concentration of visits with a core set of providers, yielding an assessment of team-based continuity

Caregiver’s survey [25]*	Questionnaire (completed by caregivers)	Questions taken from Medical Home Family Index (MHI), and the Client Perception of Coordination Questionnaire (CPCQ) Measures quality of life, satisfaction, care coordination and self-efficacy.

Care-coordination Measurement Tool (CCMT) [23]	Simple administration method (from visit records)	Records all non-reimbursable care coordination activity encounters performed by office-based personnel

Family Experiences of Coordination of Care (FECC) [26,28]	Questionnaire (completed by caregivers)	20 quality measures Questions in three main areas (care coordination services, messaging, and protocols/plans)

Framework for evaluating patient care communication [31]*† (Also measures Quality of communication)	Three stage process: 1) Questionnaire (completed by caregivers), 2) Interviews (with caregivers) and 3) Focus Groups (with HCPs)	Three-step mixed designs evaluation approach: 1) Patient quality questionnaire communication links with quality gaps (Primary Care Assessment Survey or the Measure of Processes of Care); 2) In-depth interviews with subset of patients underlying factors of quality gaps; 3) Focus group meetings with involved professionals additional related factors.

The Paediatric Integrated Care Survey (PICS) [30]† (Also measures Completeness of care)	Questionnaire (completed by caregivers)	19 experience items across 5 scales: access, communication, family impact, care goal creation and team functioning

***Outcome: Quality of collaboration***		

Collaborative Care for Attention-Deficit Disorders Scale (CCADDS) [27]	Questionnaire (completed by HCPs)	41-item instrument Measures the collaborative care processes for children with attention-deficit/hyperactivity disorder who attend primary care practices

Perception of Interprofessional Collaboration Model Questionnaire (PINCOM-Q) [34]	Questionnaire (completed by HCPs)	48-item instrument Measures perceptions of the inter-professional collaboration process on an individual, group and organisational level and includes subscales such as motivation, culture, communication, organisational aims and group leadership

Echelle De Confort Decisionnel-Partnenaire (ECD-P) [34]	Questionnaire (completed by HCPs)	Adapted version of a shared decision-making scale Focuses on the appraisal of specific clinical interactions and documenting perceptions about collaborations involving clinical events

Journey Tool [33]	Four stage process (conducted by child services evaluators)	Four phases: 1) patient case file evaluation; 2) interviews with child and caregivers about their situation and experience with the services offered; 3) meeting with associated HCPs.

Interprofessional collaboration competency scale [37]	Questionnaire (completed by health, medical, welfare and education professionals)	30-item instrument Four preliminary domains: sharing information, understanding, function, coordinating support objectives and securing

The Human Services Integration Measure Scale (HSIM) [35]	Questionnaire (completed by health, educational, social, justice, recreational, and cultural sector professionals)	5-point ordinal scale to rate both current and expected level of involvement with the other agencies within the network. Scale used to validate an integration framework which is based on awareness, communication, collaboration and cooperation

***Outcome: Continuity of care***		

Continuity of Care in Children’s Mental Health-Parent (C3MH-P) [29]	Questionnaire (completed by caregivers)	Scale based on three types of continuity: management (collaboration/transitions/flexibility), informational (information exchange/provider knowledge), and relational (interpersonal/consistency over time and transitions)

Continuity of Care in Children’s Mental Health-Youth (C3MH-Y) [29]	Questionnaire (completed by CYP)	Scale based on three types of continuity: management (collaboration/transitions/flexibility), informational (information exchange/provider knowledge), and relational (interpersonal/consistency over time and transitions)

***Outcome: Completeness of care***		

Primary Care Assessment Tool (PCATool Brazil) [36]*† (Also measures Quality of care coordination and Structure of care)	Questionnaire (completed by HCPs)	55-item Likert-scale instrument Assesses the structure and process of the system by measuring longitudinally, accessibility, coordination, system information, integrality of care and other factors

The Paediatric Integrated Care Survey (PICS) [30]† (Also measures Quality of care coordination)	Questionnaire (completed by caregivers)	19 experience items across 5 scales: access, communication, family impact, care goal creation and team functioning

***Outcome: Structure of care***		

Primary Care Assessment Instrument (PCAInstrument Brazil) [36]*† (Also measures Quality of care coordination and Completeness of care)	Questionnaire (completed by HCPs)	55-item Likert-scale instrument Assesses the structure and process of the system by measuring longitudinally, accessibility, coordination, system information, integrality of care and other factors

***Outcome: Quality of communication***		

Framework for evaluating patient care communication [31]*† (Also measures Quality of care coordination)	Three stage process: 1) Questionnaire (completed by caregivers), 2) Interviews (with caregivers) and 3) Focus Groups (with HCPs)	1) Patient quality questionnaire communication links with quality gaps (Primary Care Assessment Survey or the Measure of Processes of Care); 2) In-depth interviews with subset of patients underlying factors of quality gaps; 3) Focus group meetings with involved professionals additional related factors.

***Outcome: Local implementation of integrated care***		

Instrument to monitor the local implementation of Integrated Care for Childhood Overweight and obesity (TICCO) [32]	Questionnaire (completed by HCPs involved in project organisation)	47-item instrument Assesses eight domains: Commitment, Inter-professional teamwork, Client centeredness, Delivery system, Quality of support and care, Result-focused learning, Monitoring, and Organization and financing


*Study describes development of the measurement instrument only (not evaluated for feasibility, reliability or validity); †instrument measures more than one outcome.

The most frequent integration outcomes assessed were quality of care coordination [23,24,25,26,28,30,31,36] (measured by seven instruments) and quality of collaboration [27,33,34,35,37] (six instruments). The remaining integration outcomes assessed were: continuity of care [29] (two instruments), completeness of care [30,36] (two instruments), structure of care [36], quality of communication [31], and local implementation of integrated care [32] (each measured by one instrument, respectively). Three instruments measured more than one outcome: the Primary Care Assessment Tool – Brazil [36] (quality of care coordination, completeness of care and structure of care), the Paediatric Integrated Care Survey [30] (quality of care coordination and completeness of care), and a framework for evaluation patient care communication [31] (quality of care coordination and quality of communication).

### Evaluation of instrument measurement properties

The evaluation of the measurement properties of 13 (of the 16) instruments was conducted across 14 studies that explored a variety of measurement properties (feasibility, reliability or a type of validity). Table 2 summarises the results of the evaluation of each measurement instrument, with detailed evaluation results reported in Data file 4. Most studies found reasonable feasibility, reliability or validity (depending on the measurement property assessed), but further evaluation work is required for the Collaborative Care for Attention-Deficit Disorders Scale (CCADDS) [27] and Journey Tool [33]. All studies explored one or two measurement properties of the instrument being investigated, except Tobon et al. [29] who tested two instruments, the Continuity of Care in Children’s Mental Health-Parent (C3MH-P) and the Continuity of Care in Children’s Mental Health-Youth (C3MH-Y), for four measurement properties: reliability, and content, construct and criterion validity. The quality of study varied depending on the measurement property being evaluated. Studies were rated as ‘doubtful’ or ‘inadequate’ for the evaluation of test-retest reliability [29,30,35], and content [29,32,33] and criterion [29] validity whereas studies were rated ‘very good’ where they evaluated reliability in terms of internal consistency [27,30,34,37] and construct validity [24,27,28,29,30,37].

**Table 2:** Summary of evaluation results of instruments (n = 13) measuring integration within children and young people healthcare systems.


MEASUREMENT INSTRUMENT	FEASIBILITY	RELIABILITY	VALIDITY		

			CONTENT	CONSTRUCT	CRITERION

Care-coordination Measurement Tool (CCMT)[23]*	✓				

Bice-Boxerman Continuity of Care [24]*				✓	

Family Experiences with Coordination of Care (FECC) [26,28]	✓			✓	

Collaborative Care for Attention-Deficit Disorders Scale (CCADDS)[27]		✓		✗	

Instrument to monitor the local implementation of Integrated Care for Childhood Overweight and obesity (TICCO) [32]	✓		✓		

Perception of Interprofessional Collaboration Model Questionnaire (PINCOM-Q) [34]	✓	✓			

Echelle de confort decisionnel-partenaire (ECD-P) [34]	✓	✓			

Journey Tool [33]			✗		

Interprofessional collaboration competency scale [37]*		✓		✓	

Continuity of Care in Children’s Mental Health-Parent (C3MH-P) [29]*		✓	✓	✓	✓

Continuity of Care in Children’s Mental Health-Youth (C3MH-Y) [29]*		✓	✓	✓	✓

The Human Services Integration Measure Scale (HSIM)[35]*		✓			

The Paediatric Integrated Care Survey (PICS) [30]*		✓		✓	


✓ = reasonable feasibility (quality of methods not assessed); ✓ = reasonable reliability or validity (methods very good); ✓ = reasonable reliability or validity (methods doubtful or inadequate); ✗ = inadequate validity (methods very good); ✗ = inadequate validity (methods doubtful or inadequate). * Highlighted by authors as promising instruments based on of administration method and measurement properties, with consideration of the type of system that the instrument was designed for and evaluated in. Wider contextual factors may be considered when selecting instruments to be used in research and practice.

## Discussion

### Summary of findings

This review identified 16 instruments that measure integration within CYP healthcare systems and networks. Instruments varied in terms of administration method, with questionnaires completed by healthcare professionals being the most frequent, and measured a range of integration outcomes that, once established, can be mapped onto integration component frameworks for CYP systems. Future work to address gaps identified in this review might focus on further exploration of the measurement of completeness and structure of care, and quality of communication. To address the need to understand integration from the perspectives of different stakeholders, further development of instruments that capture perspectives of CYP and caregivers is warranted.

The evaluation of instrument measurement properties varied across the studies, and no instrument had been evaluated for all measurement properties identified in this review (i.e. feasibility, reliability, and content, construct and criterion validity). Nevertheless, this review provides a repository of available instruments for the measurement of integration in healthcare systems for CYP, allowing flexibility and a range of options that may be suitable within different contexts. The instruments identified can be used to monitor how integration is being implemented within services. Other outcomes (for example, health outcomes, quality of life, waiting times, and CYP, caregiver, and healthcare and other professionals’ perceptions of care quality, and other relevant patient reported outcomes and experiences) will be necessary to fully understand the effectiveness of integrated healthcare systems [7].

### Strengths and limitations of review

To our knowledge, this is the first systematic review to identify and appraise measurement instruments for integration specifically designed for or evaluated within CYP healthcare systems and networks. It provides an overview of instruments being used to assess integration as well as different integration outcomes being measured. This was a rapid review, therefore streamlined searching and data extraction methods were used, and searches were limited to English language studies. Comparisons across studies was challenging as there was heterogeneity in terms of health condition and outcome of integrated healthcare being assessed and, for some studies, limited description of the measurement instrument and how it is administered and used. Evaluation studies varied in quality, as judged by COSMIN [20,21,22], depending on the measurement property being assessed, although this was consistent across studies with content validity and re-test reliability evaluations considered weaker than internal consistency and construct validity evaluations.

### Implications for research and practice

This review provides details on 16 measurement instruments available for use in research and practice to monitor integration implementation in CYP healthcare systems. We expect this information will support ICS teams in selecting or adapting measurement tools which are most appropriate for their local context. Although we have identified a range of instruments that measure integration in different ways, in the following section we attempt to highlight those instruments that may be most useful in terms of administration method (i.e. Whose perspectives is the measure based on?) and proven measurement properties (i.e. Is the instrument feasible and reliable, and does it measure the desired outcome accurately?). We have also considered the type of system that the instrument was designed for and evaluated in, to provide information on whether the instrument has the potential to be used across different health conditions.

The Continuity of Care in Children’s Mental Health-Parent (C3MH-P) [29] and -Youth (C3MH-Y) [29] instruments were the most extensively evaluated instruments, showing acceptable reliability, and content, construct and criterion validity for the measurement of continuity of care. Both instruments captured the views of end users of health care systems, caregivers and young people, with C3MH-Y being the only instrument identified that collected data from children or young people. These instruments were designed to measure integration within mental health healthcare systems, so future work might explore if these could be adapted for use in other healthcare systems and include evaluations of feasibility. The Paediatric Integrated Care Survey (PICS) [30] is completed by caregivers and is designed for systems treating a broad spectrum of chronic conditions. The survey measures both quality of care coordination and completeness of care, and demonstrates acceptable reliability and construct validity, but has not been evaluated for feasibility or content validity. The Interprofessional collaboration competency scale [37] and the Human Services Integration Measure Scale (HSIM) [35] show promise for the measurement of quality of collaboration within systems designed for complex medical needs and captures perspectives from a range of professionals, including those beyond healthcare. However, further evaluation is needed for both instruments. The Care-coordination measurement tool (CCMT) [23] and Bice Boxerman [24] method are relatively simple measures that use administration records to indicate quality of coordination. These have been evaluated in systems that are designed for a broad spectrum of conditions (complex medical needs). Although further evaluation is required, these measures may provide useful additional data alongside perspectives of service users and professionals.

## Conclusion

This review identified a variety of instruments for the measurement of integration within CYP healthcare systems and networks. The standardisation of integrated care measures may allow stronger comparisons across studies and systems; however, there is a need for instruments and measures that can continue to meet the needs of specific settings, populations and conditions being studied, as well as local contexts and preferences. Future work could focus on the further understanding of measurement properties of both existing instruments and those being developed, with higher quality and more complete evaluations. Further work is needed to determine the core components of CYP integrated care and to determine which of those components are most strongly linked to care outcomes.

## Additional Files

The additional files for this article can be found as follows:

10.5334/ijic.7028.s1 Data File 1. Search strategies.

10.5334/ijic.7028.s2 Data File 2. Summary details of included studies (n = 15).

10.5334/ijic.7028.s3 Data File 3. Excluded studies and reasons for exclusion.

10.5334/ijic.7028.s4 Data File 4. Results of included evaluation studies (n = 14, one instrument evaluated in two studies).
