# Supplementary material for: Measurement Instruments for Integration within Children and Young People Healthcare Systems and Networks: A Rapid Review of the International Literature
Source: Int J Integr Care. 2023 May 23;23(2):18. doi: 10.5334/ijic.7028 (PMC10215994; doi:10.5334/ijic.7028)
Supplement: Data File 1. — Search strategies. [file ijic-23-2-7028-s1.pdf]

Data file 1 – Search strategies

Academic literature database search terms

Concept 1<br>**Integrated care**	Concept 2<br>**Child population**	Concept 3<br>**Measurement**
"collaborative care" OR "cooperative care" OR "coordinated care" OR "coordination of care" OR "cross sectoral care" OR "functional integration" OR "horizontal integration" OR "integrated care" OR "integrated service network*" OR "integration of care" OR "intersectoral care" OR "linked care" OR "seamless care" OR "service network*" OR "shared care" OR "vertical integration" OR "continuity of care" OR "care continuity"	child* OR "young people" OR youth* OR pediatric* OR paediatric*	Measure* OR instrument OR assess* OR taxonomy OR

Grey literature search terms (Open Grey and Google)

1) Integrated care children measure
2) Integrated care pediatric measure
3) Integrated care and young people and measure
4) Coordinated care children measure
5) Coordinated care pediatric measure
6) Coordinated care and young people and measure
7) Collaborative care children measure
8) Collaborative care pediatric measure
9) Collaborative care and young people and measure
