# Supplementary material for: Measurement Instruments for Integration within Children and Young People Healthcare Systems and Networks: A Rapid Review of the International Literature
Source: Int J Integr Care. 2023 May 23;23(2):18. doi: 10.5334/ijic.7028 (PMC10215994; doi:10.5334/ijic.7028)
Supplement: Data File 2. — Summary details of included studies (n = 15). [file ijic-23-2-7028-s2.pdf]

Data file 2: Summary details of included studies (n=15)

Author(s) & Year	Country	Study Design	Health Condition	Measurement Instrument(s)
Antonelli et al., (2008)	United States	Evaluation - Feasibility	Medical Complexity	Care-coordination measurement tool (CCMT)
Araujo et al., (2018)	Brazil	Development	All health conditions	Primary Care Assessment Tool (PCATool Brazil)
Arthur et al., (2018)	United States	Evaluation - Construct validity	Medical Complexity	Bice-Boxerman continuity of care (claims-based measure)
Donnelly et al., (2020)	United States	Development	Medical Complexity	Caregiver survey
Gidengil et al., (2017)	United States	Evaluation - Feasibility	Medical Complexity	Family Experiences with Coordination of Care (FECC)
Guevara et al., 2008)	United States	Evaluation – Reliability and Construct validity	Attention-Deficit Hyperactivity Disorder (ADHD)	Collaborative Care for Attention-Deficit Disorders Scale (CCADDS)
Gulmans et al., (2007)	Netherlands	Development	Cerebral Palsy	Framework for evaluating patient care communication
Koetsier et al. (2021)	Netherlands	Evaluation – Feasibility and Content validity	Overweight and obesity	Tool to monitor the local implementation of Integrated Care for Childhood Overweight and obesity (TICCO)
Parast et al., (2018)	United States	Evaluation – Construct validity	Medical Complexity	Family Experiences with Coordination of Care (FECC)
Rousseau et al., (2012)	Canada	Evaluation – Feasibility and Reliability	Mental Health	1. Perception of Interprofessional Collaboration Model Questionnaire (PINCOM-Q)<br>2. Echelle de confort decisionnel-partenaire (ECD-P)
Rutz et al., (2014)	Netherlands	Evaluation study – Content validity	Medical Complexity	Journey Tool
Shimmura and Tadaka (2018)	Japan	Evaluation – Reliability and Construct validity	Medical Complexity	Interprofessional collaboration competency scale
Tobon et al., (2013)	United States	Development and Evaluation – Reliability, Content, Construct and Criterion validity	Mental Health	1. Continuity of Care in Children's Mental Health-Parent (C3MH-P)<br>2. Continuity of Care in Children's Mental Health-Youth (C3MH-Y)

Ye et al., (2012)	Canada	Development and Evaluation - Reliability	Medical Complexity	The Human Services Integration Measure Scale (HSIM)
Ziniel et al., (2016)	United States	Evaluation – Reliability and Construct validity	Chronic Conditions	The Paediatric Integrated Care Survey (PICS)
