# Supplementary material for: Measurement Instruments for Integration within Children and Young People Healthcare Systems and Networks: A Rapid Review of the International Literature
Source: Int J Integr Care. 2023 May 23;23(2):18. doi: 10.5334/ijic.7028 (PMC10215994; doi:10.5334/ijic.7028)
Supplement: Data File 3. — Excluded studies and reasons for exclusion. [file ijic-23-2-7028-s3.pdf]

Data file 3: Excluded studies and reasons for exclusion

Population not children or young people (n=16)

Ahgren, B. and R. Axelsson (2005). "Evaluating integrated health care: a model for measurement." International journal of integrated care 5: e01-e09.

Benito, E. B., L. S. Perruca, J. J. Carramiñana, J. L. A. Torres, M. H. Pascual, I. M. Herrera and M. S.-C. Del Pozo (2018). "Scorecard of integrated care: a relevant and timely photo to help professional as in integrated clinical management." International Journal of Integrated Care (IJIC) 18: 1-2.

Berntsen, G., A. Høyem, I. Lettrem, C. Ruland, M. Rumpsfeld and D. Gammon (2018). "A person-centered integrated care quality framework, based on a qualitative study of patients' evaluation of care in light of chronic care ideals." BMC Health Serv Res 18(1): 479.

Celona, C. A. (2015). "Measuring Acuity and Patient Progress for Youth With Special Health Care Needs in Transition Care Utilizing Nursing Outcomes." Journal of Pediatric Nursing 30(5): e15-18.

Cheng, C., L. S. Franck, X. Y. Ye, S. A. Hutchinson, S. K. Lee and K. O'Brien (2019). "Evaluating the effect of Family Integrated Care on maternal stress and anxiety in neonatal intensive care units." J Reprod Infant Psychol: 1-14.

Elorriaga, K. P., M. A. González, R. G. Llinares and E. L. Linares (2018). "IEXPAC, a tool to assess patient experience." International Journal of Integrated Care (IJIC) 18: 1-2.

Feist-Wilson, S. and N. Heron (2018). "An evaluation of the clinical skills and experience within an orthopaedic Integrated Clinical Assessment and Treatment Service." BJGP Open 1(4): bjgpopen17X101217.

Flocke SA. Measuring attributes of primary care: development of a new instrument. J Fam Pract.1997;45:64–74.

Garcia-Subirats, I., M. B. Aller, I. Vargas Lorenzo and M. L. Vázquez Navarrete (2015). "[Adaptation and validation of the CCAENA(©) scale for the measurement of continuity of care between healthcare levels in Colombia and Brazil]." Gac Sanit 29(2): 88-96.

Kitchiner, D., C. Davidson and P. Bundred (1996). "Integrated care pathways: effective tools for continuous evaluation of clinical practice." J Eval Clin Pract 2(1): 65-69.

Kwan, B. M., S. Chadha, M. K. Hamer, D. Spagnolo and S. Kee (2017). "Mixed methods evaluation of a collaborative care implementation using RE-AIM." Fam Syst Health 35(3): 295-307.

McGuiness, C. and B. Sibthorpe (2003). "Development and initial validation of a measure of coordination of health care." International Journal for Quality in Health Care 15(4): 309-318.

Moe, G. C., J. E. S. Moe and A. L. Bailey (2019). "Evaluating the implementation of collaborative teams in community family practice using the Primary Care Assessment Tool." Canadian Family Physician 65(12): e515-e522.

Oldfield, B. J., N. Muñoz, N. Boshnack, R. Leavitt, M. P. McGovern, M. Villanueva, J. M. Tetrault and E. J. Edelman (2019). ""No more falling through the cracks": A qualitative study to inform measurement of integration of care of HIV and opioid use disorder." J Subst Abuse Treat 97: 28-40.

Pati, S. N., D. Porichha and K. V. Krishnamoorty (2011). "Satisfaction of leprosy patients on integrated MDT services: as evaluated by Bargarh Integrated Community Health Project (BICHP)." Indian J Lepr 83(1): 53-59.

Valentijn, P. P., F. Pereira, C. W. Sterner, H. J. M. Vrijhoef, D. Ruwaard, J. Hegbrant and G. F. M. Strippoli (2019). "Validation of the Rainbow Model of Integrated Care Measurement Tools (RMIC-MTs) in renal care for patient and care providers." PLoS One 14(9): e0222593.

Not integration measurement tool (n=44)

Antonelli R, McAllister J. (2009) Developing Care Coordination as a Critical Component of a High Performance Pediatric Health Care System: Forging a Multidisciplinary Framework for Pediatric Care Coordination. Washington, DC: The Commonwealth Fund

Bass, R. D. and C. Windle (1973). "A preliminary attempt to measure continuity of care in a community mental health center." Community Mental Health Journal 9: 53-62.

Bethell, C., C. K. Blackwell, N. Gombojav, M. B. Davis, C. Bruner and A. S. Garner (2021). "Toward measurement for a whole child health policy: Validity and national and state prevalence of the Integrated Child Risk Index." Acad Pediatr.

Cassady, C. E., B. Starfield, M. P. Hurtado, R. A. Berk, J. P. Nanda and L. A. Friedenberg (2000). "Measuring consumer experiences with primary care." Pediatrics 105(4): 998-1003.

Castillo, R. M., G. Y. Kim, K. D. Wyatt, C. M. Lohse and T. R. Hellmich (2019). "Use of an EHR-Integrated Point-of-Care Mobile Medical Photography Application in a Pediatric Emergency Department." Appl Clin Inform 10(5): 888-897.

Chen, A. Y., S. M. Schrager and R. Mangione-Smith (2012). "Quality Measures for Primary Care of Complex Pediatric Patients." Pediatrics 129(3): 433-445.

Cheng, D. R., J. Liddle, E. Mailes and M. South (2017). "Impact of an integrated electronic handover tool on pediatric junior medical staff (JMS) handover." International Journal of Medical Informatics 108: 92-96.

Chenneville, T., K. Gabbidon, H. Drake and C. Rodriguez (2019). "Comparison of the utility of the PHQ and CES-D for depression screening among youth with HIV in an integrated care setting." Journal of Affective Disorders 250: 140-144.

Church, P. T., R. E. Grunau, L. Mirea, J. Petrie, A. S. Soraisham, A. Synnes, X. Y. Ye and K. O'Brien (2020). "Family Integrated Care (FICare): Positive impact on behavioural outcomes at 18 months." Early Human Development 151: N.PAG-N.PAG.

Connor, J. A., R. C. Antonelli, C. A. O'Connell, H. Bishop Kuzdeba, C. Porter and P. A. Hickey (2018). "Measuring Care Coordination in the Pediatric Cardiology Ambulatory Setting." J Nurs Adm 48(2): 107-113.

Courtney, D. B., A. Cheung, J. Henderson, K. Bennett, M. Battaglia, J. Strauss, R. Mitchell, K. Wang and P. Szatmari (2019). "Effectiveness of an Integrated Care Pathway for Adolescents with Depression: A Pilot Clinical Trial Protocol." Journal of the Canadian Academy of Child & Adolescent Psychiatry 28(3): 115-133.

Dyke, P., P. Buttigieg, A. M. Blackmore and A. Ghose (2006). "Use of the Measure of Process of Care for families (MPOC-56) and service providers (MPOC-SP) to evaluate family-centred services in a paediatric disability setting." Child: Care, Health & Development 32(2): 167-176.

Ginsburg, A. D., P. S. Stadem, C. R. Takala, P. E. Croarkin, A. B. Mattson, M. L. Billings, R. M. Brennan and J. E. Huxsahl (2018). "An Examination of Screening Tools for Collaborative Care of Adolescent Depression." J Clin Psychiatry 79(4).

Gulmans, J., M. M. Vollenbroek-Hutten, J. E. Van Gemert-Pijnen, W. H. Van Harten, J. Gulmans, M. M. R. Vollenbroek-Hutten, J. E. W. C. Van Gemert-Pijnen and W. H. Van Harten (2009). "Evaluating patient care communication in integrated care settings: application of a mixed method approach in cerebral palsy programs." International Journal for Quality in Health Care 21(1): 58-65.

Gunda, A., A. Jousset, T. Tchereni, J. Joseph and V. Mwapasa (2017). "Integrating HIV and Maternal, Neonatal and Child Health Services in Rural Malawi: An Evaluation of the Implementation Processes and Challenges." J Acquir Immune Defic Syndr 75 Suppl 2: S132-s139.

Hewes, H. A., M. Ely, R. Richards, M. I. Shah, S. Busch, D. Pilkey, K. D. Hert and L. M. Olson (2019). "Ready for Children: Assessing Pediatric Care Coordination and Psychomotor Skills Evaluation in the Prehospital Setting." Prehospital Emergency Care 23(4): 510-518.

Khetani, M. A., A. B. Cliff, C. Schelly, L. Daunhauer and D. Anaby (2014). "Decisional Support Algorithm for Collaborative Care Planning using the Participation and Environment Measure for Children and Youth (PEM-CY): A Mixed Methods Study." Phys Occup Ther Pediatr.

Knapp, C., V. Madden, P. Sloyer and E. Shenkman (2012). "Effects of an Integrated Care System on Quality of Care and Satisfaction for Children with Special Health Care Needs." Maternal & Child Health Journal 16(3): 579-586.

Kolko, D. J., J. Campo, A. M. Kilbourne, J. Hart, D. Sakolsky and S. Wisniewski (2014). "Collaborative care outcomes for pediatric behavioral health problems: a cluster randomized trial." Pediatrics 133(4): e981-992.

Lawson, K., S. Bloom, M. Sadof, C. Stille and J. Perrin (2011). "Care Coordination for Children with Special Health Care Needs: Evaluation of a State Experiment." Maternal & Child Health Journal 15(7): 993-1000.

Lynch, S., C. Greeno, J. L. Teich and J. Heekin (2019). "Pediatric integrated behavioral health service delivery models: Using a federal framework to assess levels of integration." Social Work in Health Care 58(1): 32-59.

Marcu, M. I., C. A. Knapp, D. Brown, V. L. Madden and H. Wang (2016). "Assessing the impact of an integrated care system on the healthcare expenditures of children with special healthcare needs." Am J Manag Care 22(4): 272-280.

Marcu, M. I., C. A. Knapp, V. L. Madden, H. Wang, M. Kaufmann and P. Sloyer (2014). "Effect of an integrated care system on utilization for CSHCN in Florida." Matern Child Health J 18(1): 38-44.

Miller, E., B. Smith, K. Costantino and J. Eastwood (2017). ""Instead of just assuming what people need, actually ask them what they're after": Patient Reported Measures for families with complex needs." International Journal of Integrated Care (IJIC) 17: 1-2.

Newham, J. J., J. Forman, M. Heys, S. Cousens, C. Lemer, M. Elsherbiny, R. M. Satherley, R. Lingam and I. Wolfe (2019). "Children and Young People's Health Partnership (CYPHP) Evelina London model

of care: protocol for an opportunistic cluster randomised controlled trial (cRCT) to assess child health outcomes, healthcare quality and health service use." BMJ Open 9(8): e027301.

O'Brien, K., K. Robson, M. Bracht, M. Cruz, K. Lui, R. Alvaro, O. da Silva, L. Monterrosa, M. Narvey, E. Ng, A. Soraisham, X. Y. Ye, L. Mirea, W. Tarnow-Mordi and S. K. Lee (2018). "Effectiveness of Family Integrated Care in neonatal intensive care units on infant and parent outcomes: a multicentre, multinational, cluster-randomised controlled trial." Lancet Child Adolesc Health 2(4): 245-254.

Palfrey, J. S., L. A. Sofis, E. J. Davidson, J. Liu, L. Freeman and M. L. Ganz (2004). "The pediatric alliance for coordinated care: evaluation of a medical home model." Pediatrics 113(5 Part 2): 1507-1516.

Patel, P. H., C. Welsh and M. B. Foggs (2004). "Improved asthma outcomes using a coordinated care approach in a large medical group." Disease Management 7(2): 102-111.

Ponnet, L., S. Willems, V. Vyncke, A. E. M. Bousquat, A. L. D. Viana, G. A. Mello and M. Demarzo (2019). "Evaluation of the quality of Primary Health Care services for children: reflections on the feasibility of using the Brazilian version of the Primary Care Assessment Tool as a routine assessment tool." Einstein (Sao Paulo) 17(1): eAO4333.

Rattler, T. L., A. M. Walder, H. Feng and J. L. Raphael (2016). "Care Coordination for Children With Sickle Cell Disease: A Longitudinal Study of Parent Perspectives and Acute Care Utilization." American Journal of Preventive Medicine 51: S55-S61.

Reiss, M., C. A. Greene and J. D. Ford (2017). "Is it time to talk? Understanding specialty child mental healthcare providers' decisions to engage in interdisciplinary communication with pediatricians." Social Science & Medicine 175: 66-71.

Rucci, P., J. Latour, E. Zanello, S. Calugi, S. Vandini, G. Faldella and M. P. Fantini (2015). "Measuring parents' perspective on continuity of care in children with special health care needs." Int J Integr Care 15: e046.

Saleh, M. and N. A. Almasri (2014). "Use of the Measure of Processes of Care (MPOC-20) to evaluate health service delivery for children with cerebral palsy and their families in Jordan: validation of Arabic-translated version (AR- MPOC-20)." Child: Care, Health & Development 40(5): 680-688.

Schmidt, S., U. Thyen, C. Herrmann-Garitz, F. Bomba and H. Muehlan (2016). "The Youth Health Care measure-satisfaction, utilization, and needs (YHC-SUN)-development of a self-report version of the Child Health Care (CHC-SUN) proxy-measure." BMC Health Services Research 16: 1-12.

Shippee, N. D., A. Mattson, R. Brennan, J. Huxsahl, M. L. Billings and M. D. Williams (2018). "Effectiveness in Regular Practice of Collaborative Care for Depression Among Adolescents: A Retrospective Cohort Study." Psychiatric Services 69(5): 536-541.

Siden, H., L. Straatman, T. Miller and J. Ham (2009). "The Madison Clinic: Evaluation of a collaborative outpatient paediatric palliative care clinic." Paediatr Child Health 14(6): 379-384.

Srinivasan, R., A. Marathe, A. A. Sane and V. Krishnamurthy (2022). "Adaptation of the Measure of Processes of Care for the Evaluation of Family-Centeredness of Services in India." J Dev Behav Pediatr 43(2): e79-e86.

Stadnick, N. A., G. A. Aarons, K. Martinez, M. Sklar, K. J. Coleman, D. P. Gizzo, E. Lane, C. L. Kuelbs and L. Brookman-Frazee (2022). "Implementation outcomes from a pilot of "Access to Tailored

Autism Integrated Care" for children with autism and mental health needs." Autism: 13623613211065801.

Suen, C. G., K. Campbell, G. Stoddard and P. S. Carbone (2020). "Patient-Centered Outcomes in an Interdisciplinary Clinic for Complex Children with Autism." J Dev Behav Pediatr.

Sultan, M. A., C. S. Pastrana and K. A. Pajer (2018). "Shared Care Models in the Treatment of Pediatric Attention-Deficit/Hyperactivity Disorder (ADHD): Are They Effective?" Health Serv Res Manag Epidemiol 5: 2333392818762886.

Williams, L. J., K. Waller, R. P. Chenoweth and A. L. Ersig (2020). "Stakeholder perspectives: Communication, care coordination, and transitions in care for children with medical complexity." J Spec Pediatr Nurs: e12314.

Wood, D. L., Q. E. McCaskill, N. Winterbauer, E. Jobli, T. Hou, P. Wludyka, K. Stowers and W. Livingood (2009). "A multi-method assessment of satisfaction with services in the medical home by parents of children and youth with special health care needs (CYSHCN)." Maternal & Child Health Journal 13(1): 5-17.

Zanello, E., S. Calugi, L. M. Sanders, J. Lenzi, G. Faldella, P. Rucci and M. P. Fantini (2017). "Care coordination for children with special health care needs: a cohort study." Italian Journal of Pediatrics 43: 1-6.

Zima, B. T., M. McCreary, K. Kenan, M. Churchey-Mims, H. Chi, M. Brady, J. Davies, V. Rompala and B. Leventhal (2018). "Development and Evaluation of Two Integrated Care Models for Children Using a Partnered Formative Evaluation Approach." Ethnicity & Disease 28: 445-456.

Not development or evaluation study (n=7)

Agency for Healthcare Research and Quality. (2011). Appendix IV. Care Coordination Measure Instruments. Care Coordination Measures Atlas. Rockville, MD: U.S. Department of Health and Human Services.

Center for Medical Home Improvement. (2001). The Medical Home Family Index survey. Concord, NH: Crotched Mountain Foundation.

Christakis, D. A., J. A. Wright, F. J. Zimmerman, A. L. Bassett and F. A. Connell (2003). "Continuity of care is associated with well-coordinated care." Ambulatory Pediatrics 3(2): 82-86.

Liersch, S., K. Krüger, C. Oedingen, A. Spreenberg, T. Bergemann and C. Krauth (2020). "Evaluation of the pediatric-centered integrated care AOK Junior: protocol for a mixed-method study." BMC Health Services Research 20(1): 1-10.

Lyles, A. A., P. M. Overgaard, G. L. Caputo and E. Reifsnider (2017). "Stakeholders' Perceptions of Care Coordination: A Participatory Process." Journal of Pediatric Healthcare 31(5): 555-559.

Sheftall, A. H., D. J. Chisolm, E. R. Alexy, L. J. Chavez, R. M. Mangione-Smith, R. M. Ferrari and P. H. Song (2019). "Satisfaction With Care Coordination for Families of Children With Disabilities." Journal of Pediatric Healthcare 33(3): 255-262.

Ye, C., G. Browne, V. S. Grdisa, J. Beyene and L. Thabane (2012). "Measuring the degree of integration for an integrated service network." Int J Integr Care 12: e137.

Not full primary study (n=17)

Callejo-Black, A., D. V. Wagner, K. Ramanujam, A. J. Manabat, S. Mastel and A. R. Riley (2020). "A Systematic Review of External Validity in Pediatric Integrated Primary Care Trials." J Pediatr Psychol 45(9): 1039-1052.

Cohen, E. and R. J. Coller (2020). "Evaluating Integrated Care for Children: A Clarion Call or a Call for Clarity?" Pediatrics 145(1): 1-2.

Eastwood, J., S. Woolfenden, E. Miller, M. Shaw, P. Garg, H. Liu, D. De Souza and R. Ettema (2019). "Evaluating the implementation, mechanisms of effect and context of an integrated care intervention for vulnerable families in Central Sydney Australia: A research framework." International Journal of Integrated Care (IJIC) 19(S1): 1-2.

Ewing, C. I., S. A. Cropper and T. B. Horsburgh (2016). "Developing, implementing and evaluating integrated care models for infants, children, young people and their families." Archives of Disease in Childhood 101(9): 781-782.

Fogel, B., D. Waschbusch, B. Jairath, D. Sekhar and J. Waxmonsky (2019). "Feasibility and effectiveness of implementing an integrated care model for attention deficit hyperactivity disorder in primary care pediatrics." International Journal of Integrated Care (IJIC) 19(S1): 1-2.

Greenbaum PE, Lipien L, Dedrick RF. An instrument to measure inter-agency collaboration among child-serving organizations. Paper presented at: American Psychological Association, 2004; Honolulu, Hawaii.

Jeffrey, J. K., M. Sinclair, H. Aralis, R. Linonis, W. Barrera, A. Semaan, M. Grossman and P. Lester (2017). "6.60 Acceptability and Results of Web-Based Behavioral Health Assessment Platform Implemented Within a Primary Care Pediatric Integrated Care Clinic." Journal of the American Academy of Child & Adolescent Psychiatry 56: S297-S297.

Jorina, M., A. Cammaerts, J. Singer, A. Ozonoff, E. Chan, R. Antonelli, M. Gurvitz and K. Jenkins (2016). "Advancing the Measurement of Care Coordination in Pediatric Behavioral Health." J Dev Behav Pediatr 37(8): 674-684.

Lingam, R., J. Forman, J. Newham, S. Cousens, R.-M. Satherley, M. El Sherbiny and I. Wolfe (2019). "The Children and Young People's Health Partnership (CYPHP) Evelina London Model of Care: an opportunistic cluster randomised trial to assess child health outcomes, healthcare quality, and health service use." International Journal of Integrated Care (IJIC) 19(S1): 1-2.

McLeigh, J. D., et al. (2022). "Pediatric integrated care in the primary care setting: A scoping review of populations served, models used, and outcomes measured." Child: Care, Health and Development, 1– 11

Moss, A. and R. Miller (2019). "Models of community based integrated care for people with a learning disability and/or autism: evaluation findings from a national implementation programme." International Journal of Integrated Care (IJIC) 19(S1): 1-2.

Pandey, A., A. Gireesh and R. Viner (2019). "Feasibility, acceptability, and effectiveness of young people-specific, integrated out-of-hospital services: a protocol for a systematic review." Syst Rev 8(1): 77.

Sarvet, B. (2020). "Measurement-Based Care in the Pediatric Primary Care Setting." Child Adolesc Psychiatr Clin N Am 29(4): 691-702.

Starfield, B. H., D. W. Simborg, S. D. Horn and S. A. Yourtee (1976). "Continuity and coordination in primary care: their achievement and utility." Medical Care 14: 625-636.

Sternberg S, Co J, Homer C. Review of quality measures of themost integrated health care settings for children and the need forimproved measures: recommendations for initial coremeasurement set for CHIPRA.Acad Pediatr.2011;11(3 suppl): S49–S58.

Suter, E., N. Oelke, M. A. Lima, M. Stiphout, R. Janke, R. Witt, C. Van Vliet-Brown, K. Schill, M. Rostami, S. Hepp, A. Birney, F. Al-Roubaiai and G. Marques (2017). "Indicators and Measurement Tools for Health Systems Integration: A Knowledge Synthesis." International Journal of Integrated Care 17.

Zurynski, Y., L. Altman, C. Breen and S. Woolfenden (2018). "An evaluation of care coordination for children with chronic and complex conditions in Australia: experiences and needs of healthcare providers." International Journal of Integrated Care (IJIC) 18: 1-2
