# Supplementary material for: Measurement Instruments for Integration within Children and Young People Healthcare Systems and Networks: A Rapid Review of the International Literature
Source: Int J Integr Care. 2023 May 23;23(2):18. doi: 10.5334/ijic.7028 (PMC10215994; doi:10.5334/ijic.7028)
Supplement: Data File 4. — Results of included evaluation studies (n = 14, one instrument evaluated in two studies). [file ijic-23-2-7028-s4.pdf]

Data file 4: Results of included evaluation studies (n=14, one instrument evaluated in two studies)

Author(s) & Year	Study Design	Measurement Instrument	Evaluation results
Antonelli et al., (2008)	Evaluation - Feasibility	Care-coordination measurement tool (CCMT)	Care-coordination can be assessed at the practice level, as well as across multiple practices. CCMT can be successfully used during the day-to-day operations in a variety of pediatric, primary care settings.
Arthur et al., (2018)	Evaluation - Construct validity	Bice-Boxerman continuity of care (claims-based measure)	Higher continuity of care scores were associated with lower odds of emergency department utilization and higher probability that caregivers reported receiving needed care coordination services. Results are consistent with the hypothesis that higher continuity of care would be associated with lower emergency department utilization and better care coordination outcomes. There was no significant association between continuity of care and satisfaction with care coordination or lower effects on family related to coordinating their child's care.
Gidengil et al., (2017)	Evaluation - Feasibility	Family Experiences with Coordination of Care (FECC)	The FECC survey enables the evaluation of care coordination quality for CMC. Both survey modes (telephone only and mailing plus telephone) were feasible to implement, but mailing plus telephone administration produced a higher response rate.
Parast et al., (2018)	Evaluation – Construct validity	Family Experiences with Coordination of Care (FECC)	Nineteen of the twenty FECC quality measures were significantly and positively associated with one or more of the validation measures. The components of care coordination demonstrating the strongest positive association with provider ratings included (1) having a care coordinator who was knowledgeable, supportive and advocated for the child's needs (β =26.4; 95% Confidence Interval [CI] 20.0,32.8, scaled to reflect change associated with a 0 to 100 change in the FECC measure score) and (2) receiving a written visit summary that was useful and easy to understand (β =22.0; 95% CI 17.1,27.0).<br>Nineteen newly developed FECC quality measures demonstrated convergent validity with previously validated CAHPS measures. These new measures are valid for assessing the quality of care coordination services provided to CMC and may be useful for evaluating new models of care focused on improving these services.
Guevara et al., 2008)	Evaluation – Construct validity and Reliability	Collaborative Care for Attention-Deficit Disorders Scale (CCADDS)	Internal consistency reliability for the overall scale was 0.91, and subscale scores ranged from 0.80 to 0.89. The CCADDS correlated with a validated

			measure of provider psychosocial orientation (r= -0.36, P<0.001) and with self-reported frequency of mental health referrals or consultations (r= -0.24 to -0.42, P<0.001). CCADDS scores were similar among physicians by race/ethnicity, gender, age group, and practice location.<br>Scores on the CCADDS were reliable for measuring collaborative care processes in this sample of primary care clinicians who provide treatment for children with attention-deficit/hyperactivity disorder. Evidence for validity of scores was limited.
Koetsier et al. (2021)	Evaluation – Feasibility and Content validity	Tool to monitor the local implementation of Integrated Care for Childhood Overweight and obesity (TICCO)	The results from the pilot study with the preliminary tool showed individual differences in element scores within municipalities. Local discussion on the interpretation of individual TICCO scores could raise multiple opportunities for the further development of these local approaches. Therefore, the TICCO is considered to be an appropriate practice-based local evaluation and discussion tool for individual TICCO scores, rather than being a rigorously validated measurement tool to monitor the local implementation of integrated care for childhood overweight and obesity. The TICCO tool appears to be most suitable for those operating in a central position within integrated care practice, such as coordinating professionals and project leaders.
Rousseau et al., (2012)	Evaluation – Feasibility and Reliability	Perception of Interprofessional Collaboration Model Questionnaire (PINCOM-Q)	Internal consistency estimates of reliability computed for the PINCOM-Q gave values for Cronbach Alpha of 0.94.
Rousseau et al., (2012)	Evaluation – Feasibility and Reliability	Echelle de confort decisionnel-partenaire (ECD-P)	Internal consistency estimates of reliability computed for the ECD-P gave values for Cronbach Alpha of 0.93.
Rutz et al., (2014)	Evaluation study – Content validity	Journey Tool	Through analysis of 24 "journeys of care", inspectors used only one form of coordination - the creation of hierarchy resulting in one problem definition. But in complex care practices, children have multiple and often incompatible problems so one coherent problem definition cannot be made.<br>To improve the journey tool to stimulate professionals and organizations to create better childcare outcomes, it is necessary that inspectors broaden the scope of their assessments. The patchwork design of the journey tool facilitates inspectors to look into the multiplicities and diversity of complex care practices and to reflect on the results for children.
Shimmura and Tadaka (2018)	Evaluation – Reliability and Construct validity	Interprofessional collaboration competency scale	Exploratory and confirmatory factor analyses identified 12 items on three factors: ‘sharing needs assessment skills’, ‘resource development skills’ and

			'creative networking skills'. The final model showed good fit on four indices (eg, goodness of fit index: 0.925). The Cronbach's alpha for the entire scale was 0.93 and was above 0.80 for each factor. The correlation coefficient between the existing scale and the ICC-CMC was 0.72 (p<0.001).<br>The ICC-CMC demonstrated acceptable internal consistency and validity.
Tobon et al., (2013)	Development and Evaluation – Reliability, Content, Construct and Criterion validity	Continuity of Care in Children's Mental Health-Parent (C3MH-P)	Reliability: Internal consistencies (Cronbach's alpha) ranged from 0.80 to 0.93. The C3MH-P demonstrated good test–retest reliability with ICCs for the five scales ranging from 0.75 to 0.92 with no differences in scale means between administrations.<br>Construct validity: Higher satisfaction with services (CSQ scores) was significantly correlated with all five scales on the C3MH-P. There was weak, and significantly negative, correlation between parental depression and the transitions scale but no other significant correlations with the DASS-21 subscale. There was a weak, but not statistically significant, correlation between length of time in treatment the provider knowledge scale. The relational continuity scales were moderately to strongly significantly correlated with therapeutic alliance. There were no significant correlations between barriers and continuity. Higher child internalizing problems was weakly but significantly positively correlated with the provider knowledge and relational consistency scales. Higher child externalizing problems was weakly but significantly negatively correlated with the transitions and relational: interpersonal scales. Impact of child problems on the family was negatively correlated with the transitions and relational: interpersonal scales. Total child problems and greater child impairment were negatively correlated with the transitions scale.<br>Criterion validity: There were small to moderate significant differences between the case management groups on three of the five C3MH-P scales: provider knowledge, relational: consistency and transitions. There were no significant differences between the case management groups in terms of satisfaction. There were significant differences between the drop-out groups on all of the C3MH-P scales and in terms of satisfaction.
Tobon et al., (2013)	Development and Evaluation – Reliability, Content, Construct and Criterion validity	Continuity of Care in Children's Mental Health-Youth (C3MH-Y)	Reliability: The internal consistency of the C3MH-Y was reasonable for all three scales.<br>Construct validity: For the C3MH-Y, higher satisfaction with services was significantly correlated with management continuity (r=0.71), informational continuity (r=0.58) and relational continuity (r=0.84). The relational

			continuity scale was significantly correlated with therapeutic alliance (r=0.70).<br>Criterion validity: For the C3MH-Y, there were no significant differences between those with or without a case manager on any of the scales, range of d (standardized effect size) = 0.06–0.47. Youth who dropped out of treatment, versus those who did not, had significantly lower scores on management, t(9.52)=3.03, p=0.013, d=0.86, and relational continuity t(53)=2.99, p=0.004, d=0.87, but not informational continuity, t(54)=1.66, p=0.10, d=0.45.
Ye et al., (2012)	Development and Evaluation - Reliability	The Human Services Integration Measure Scale (HSIM)	Most agencies' integration scores were <65%. As measured by the agreement between every other agency's perception and expectation, the overall integration of CTN in Simcoe and York was 44% (95% CI: 39%–49%) and 52% (95% CI: 48%–56%), respectively. The sensitivity analysis showed that the global scores were robust.
Ziniel et al., (2016)	Evaluation – Reliability and Construct validity	The Paediatric Integrated Care Survey (PICS)	Cronbach's α values were close to or >0.7 for 4 of the 5 constructs. Internal consistency was also established through strong correlation coefficients with values ≥0.4 between items and their own composite for 4 of the 5 constructs. Three family impact items showed moderate or weak correlation coefficients (≤0.33). However, the range and mean of the correlation coefficients between items and the other composite scales were always lower than those between the items and their own composite scale, demonstrating strong discriminant validity. With regard to test–retest reliability, 10 items agreed substantially between the 2 survey administrations, with κ values >0.60. The other 9 items showed moderate agreement, with κ values between 0.41 and 0.60<br>Nearly all health care needs and usage indicators show lower composite scores for those indicating higher needs or usage. Although the differences were significant for the composite scales access and team functioning, only about one-half of the indicators for the composite scales of communication and family impact reached statistical significance. However, differences in the mean scores were directionally as expected. If all health care providers had access to the same medical records, composite scores were, as expected, significantly higher for these 4 domains. For the composite scale of care goal creation, none of the groups exhibited significant differences, and the direction of the composite score means was not always as expected. Having services from allied health professionals, medical

			equipment, and using >2 health care providers showed increased mean scores. Parents of children using behavioral health care showed significantly lower mean scores on all composites and most individual items. Care received from providers within 1 health system was rated significantly higher with regard to 4 composites and most of the related items. The only composite showing no difference between the health care systems was care goal creation. Insurance type did not show any significant differences for composite and most individual item ratings.<br>The final survey contained 19 experience items in 5 scales: access, communication, family impact, care goal creation, and team functioning. Psychometric analyses supported these 5 scales.
